# Supplementary material for: Ionic Liquid-Modulated Synthesis of Porous Worm-Like Gold with Strong SERS Response and Superior Catalytic Activities
Source: Nanomaterials (Basel). 2019 Dec 12;9(12):1772. doi: 10.3390/nano9121772 (PMC6955750; doi:10.3390/nano9121772)
Supplement: Supplementary file 1 [file nanomaterials-09-01772-s001.pdf]

nanomaterials

Supporting Information

Article

Ionic Liquid-Modulated Synthesis of Porous Worm-Like Gold with Strong SERS Response and Superior Catalytic Activities

Kaisheng Yao [1,*], Nan Wang [1], Zhiyong Li [2], Weiwei Lu [1] and Jianji Wang [2,*]

[1] School of Chemical Engineering and Pharmaceutics, Henan University of Science and Technology, Luoyang 471023, Henan, China; 18848962198@163.com (N.W.); luwei1980@126.com (W.L.)

[2] Collaborative Innovation Center of Henan Province for Green Manufacturing of Fine Chemicals, Key Laboratory of Green Chemical Media and Reactions, Ministry of Education, School of Chemistry and Chemical Engineering, Henan Normal University, Xinxiang 453007, Henan, China; lizhiyong03@126.com

* Correspondence: ksyao@126.com (K.Y.); jwang@htu.cn (J.W.)

Received: 14 November 2019; Accepted: 10 December 2019; Published: date

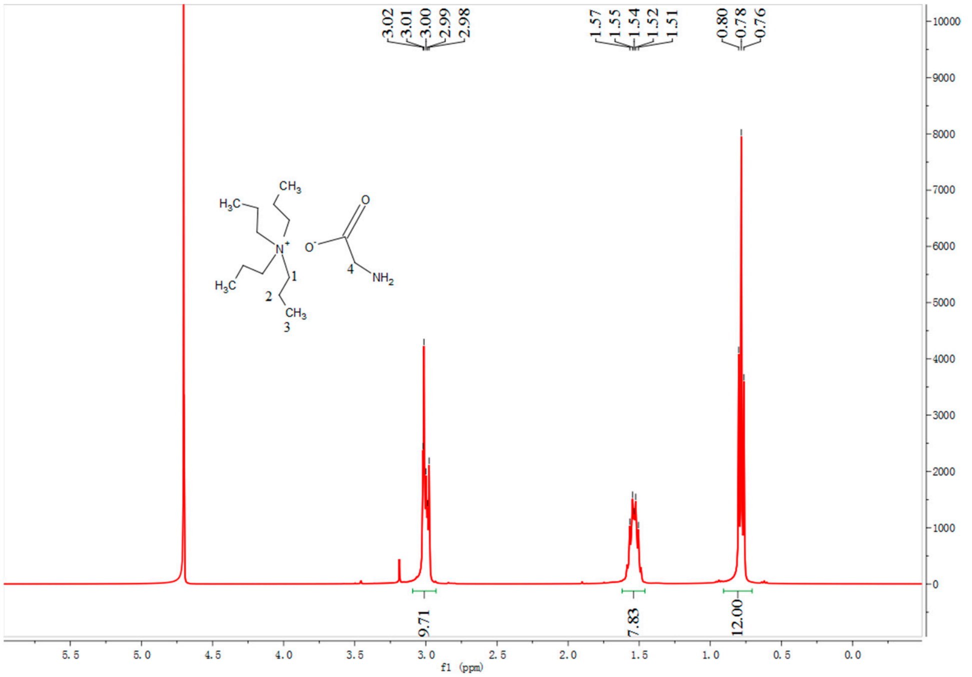

Figure S1. ^{1}H NMR spectra of synthesized ionic liquid [N_{3333}][Gly]. ^{1}H NMR (400 MHz, D_2O) δ 3.09 - 2.93 (m, 10H, H(1) and (4)), 1.62 - 1.46 (m, 8H, H(2)), 0.78 (t, J = 7.3 Hz, 12H, H(3)).

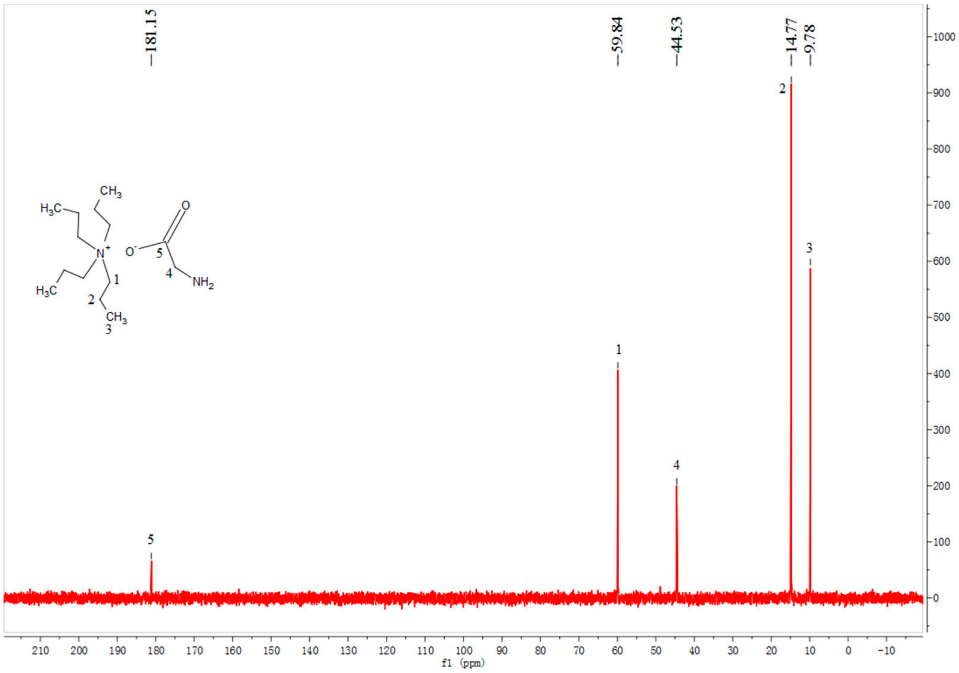

Figure S2. ^{13}C NMR spectra of synthesized ionic liquid [N3333][Gly]. ^{13}C NMR (101 MHz, D_2O) δ 181.15 (s, C(5)), 59.84 (s, C(1)), 44.53 (s, C(4)), 14.77 (s, (2)), 9.78 (s, C(3)).

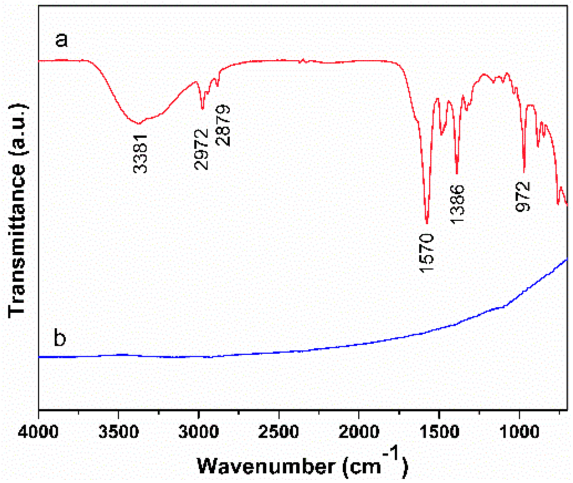

Figure S3. FTIR spectra of (**a**) [N3333][Gly] and (**b**) porous Au worms.

Figure S3 a presents the FTIR spectra of synthesized [N_{3333}][Gly]. The wide peak at 3381 cm^{-1} is attributed to $–NH_2$ asymmetric telescopic vibration. The peaks at 2972 and 2879 cm^{-1} belong to asymmetric and symmetric stretching vibration of $–CH_3$, respectively. The peak at 1570 cm^{-1} is the characteristic absorption of $–COO^-$. The band at 1386 cm^{-1} is assigned to the symmetric variable angle vibration of $–CH_3$. The 972 cm^{-1} is ascribed to the C–N bending vibration in quaternary ammonium ions.

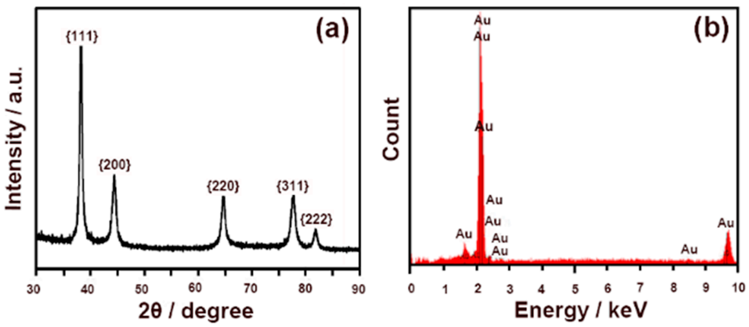

Figure S4. (a) XRD pattern and (**b**) EDX spectrum of porous Au worms synthesized in the presence of [N3333][Gly].

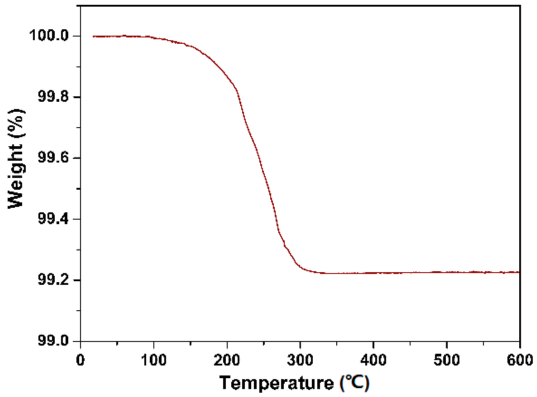

Figure S5. TG curve of porous Au worms.

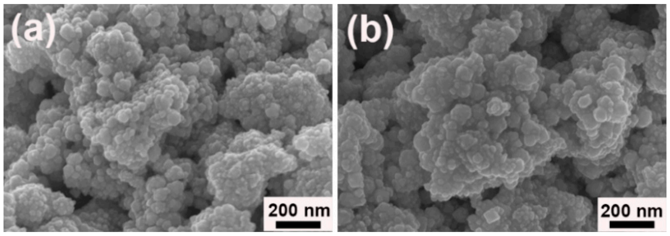

Figure S6. FESEM images of Au samples synthesized with different ascorbic acid concentrations: (**a**) 50 mM, and (**b**) 200 mM.

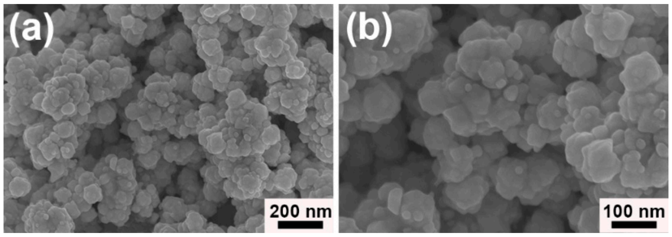

Figure S7. FESEM images of Au samples synthesized in the presence of [N3333][Gly] under vigorous stirring.

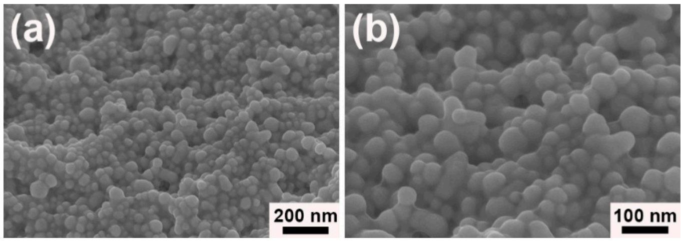

Figure S8. FESEM images of the Au samples synthesized in the absence of [N3333][Gly].

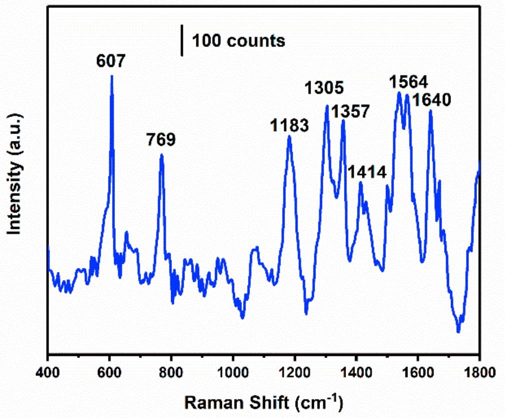

Figure S9. The normal Raman spectrum of pure solid R6G.

The Calculation of SERS Enhancement factor (EF):

The EF was defined as follows [1,2]

$$EF = (I_{SERS}/N_{ads}) \,/\, (I_{bulk}/N_{bulk}) \qquad \text{(S1)}$$

where I_{SERS} and I_{bulk} denote the Raman intensity of R6G in SERS and normal Raman spectrum, respectively. For the calculation of EF values, the peak intensity at 1358 cm^{-1} was used. N_{ads} and N_{bulk} are the number of R6G molecules adsorbed on the SERS substrates and number of bulk molecules within the SERS detecting spot, respectively.

N_{ads} could be calculated using the following equation [1]:

$$N_{ads} = N_d A_{laser} A_N/\sigma \qquad \text{(S2)}$$

where N_d is the number density of porous worm-like Au, A_{laser} is the area of the focal spot of laser, A_N is the footprint area of Au product, and σ is the surface area occupied by an adsorbed R6G molecule on full coverage of Au, which is about 4 nm^2 [1,2]. Due to the complex geometry, it is difficult to calculate the accurate surface area of porous Au worms. It is assumed that porous worm-like Au has a compact flat surface. In addition, the spot diameter of the laser beam is about 1 μm in our experiment. Thus, the total number of N_{ads} within the laser spot was calculated to be about 1.96×10^5. N_{bulk} is the molecule number of the solid R6G in the laser illumination volume. The penetration depth of laser spot was about 2 μm and the solid R6G density was 1.26 g/cm^3 [1]. Then, N_{bulk} of R6G was calculated to be 2.49×10^9. Finally, according to the equation (S1), the EF was calculated to be 3.5×10^6.

References

1. Wang, W.; Han, Y.; Gao, M.; Wang, Y. Facile synthesis of two-dimensional highly branched gold nanostructures in aqueous solutions of cationic gemini surfactant. *CrystEngComm.* **2013**, *15,* 2648–2656.
2. Sun, N.; Yao, K.; Wang, C.; Zhao, C.; Lu, W.; Zhao, S.; Wang, H.; Wang, J. Synthesis of various gold hierarchical architectures assisted by functionalized ionic liquids in aqueous solutions and their efficient SERS responses. *J. Colloid Interface Sci.* **2018**, *531*, 194–203.

© 2019 by the authors. Submitted for possible open access publication under the terms and conditions of the Creative Commons Attribution (CC BY) license (http://creativecommons.org/licenses/by/4.0/).
